# Short and Long-Term Effects of *Baccharis articulata* on Glucose Homeostasis

**DOI:** 10.3390/molecules17066754

**Published:** 2012-06-04

**Authors:** Virginia D. Kappel, Danielle F. Pereira, Luisa H. Cazarolli, Samantha M. Guesser, Carlos H. Blum da Silva, Eloir P. Schenkel, Flávio H. Reginatto, Fátima R. M. B. Silva

**Affiliations:** 1 Departamento de Bioquímica, Centro de Ciências Biológicas, Universidade Federal de Santa Catarina, Cx. Postal 5069, CEP 88040-970, Florianópolis, SC, Brazil; Email: virginiadkappel@yahoo.com.br (V.D.K.); lele.farm@hotmail.com (D.F.P.); samguesser@gmail.com (S.M.G.); 2 Universidade Federal da Fronteira Sul, Campus Universitário Laranjeiras do Sul, Bairro Vila Alberti, CEP 85303-775, Laranjeiras do Sul, PR, Brazil; Email: luisacazarolli@gmail.com; 3 Departamento de Ciências Farmacêuticas, Centro de Ciências da Saúde, Universidade Federal de Santa Catarina, Florianópolis, SC, Brazil; Email: carloshfarmacia@yahoo.com.br (C.H.B.S.); eloirschenkel@gmail.com (E.P.S.); freginatto@hotmail.com (F.H.R.)

**Keywords:** *Baccharis articulata*, diabetes, insulin, disaccharidase, glycation

## Abstract

In this study, the *in vivo* effect of the crude extract and *n*-butanol and aqueous residual fractions of *Baccharis articulata* (Lam.) Pers. on serum glucose levels, insulin secretion and liver and muscle glycogen content, as well as *in vitro* action on serum intestinal disaccharidase activity and albumin glycation were investigated. Oral administration of the extract and fractions reduced glycemia in hyperglycemic rats. Additionally, the *n*-butanol fraction, which has high flavonoids content, stimulated insulin secretion, exhibiting an insulinogenic index similar to that of glipizide. Also, the *n*-butanol fraction treatment significantly increased glycogen content in both liver and muscle tissue. *In vitro* incubation with the crude extract and *n*-butanol and aqueous residual fractions inhibited maltase activity and the formation of advanced glycation end-products (AGEs). Thus, the results demonstrated that *B. articulata* exhibits a significant antihyperglycemic and insulin-secretagogue role. These effects on the regulation of glucose homeostasis observed for *B. articulata* indicate potential anti-diabetic properties.

## 1. Introduction

Diabetes mellitus (DM) is a chronic metabolic disorder characterized by a high blood glucose concentration (hyperglycemia) which is due to insulin deficiency and/or insulin resistance. As a consequence, liver and skeletal muscle is unable to take up or utilize glucose or to store it as glycogen. The chronic hyperglycemia of diabetes is associated with long-term damage to many systems of the body, in particular the eyes, kidneys, nerves, heart, and blood vessels [1]. 

Type 2 diabetes mellitus (T2DM) is the most common metabolic disorder worldwide, and with epidemic proportions the increase in its prevalence is unprecedented, both in developed and developing countries. The primary aim in the management of T2DM is to delay, or even prevent, the complications of the disease by achieving euglycemic levels. In addition to drug therapy, with oral hypoglycemic agents and/or insulin, the glycemic control often involves changes in lifestyle such as diet and amount of exercise. However, the treatment of T2DM is complicated by several factors inherent to the disease process, such as insulin resistance, hyperinsulinemia, impaired insulin secretion and reduced insulin-mediated glucose uptake and utilization [2].

A variety of plant extracts have been used for centuries in folk medicine to treat diabetes. Medicinal plants are particularly interesting since not only can they be used as complementary and alternative remedies to prevent metabolic diseases, but they also serve as an interesting source of compounds which are potential drug candidates [3]. Several plant species have demonstrated anti-diabetic properties and a large number of compounds from plant extracts have been reported to have beneficial effects in the treatment of diabetes [4].

The *Baccharis* genus (Asteraceae) is distributed mainly in Brazil, Argentina, Colombia, Chile and Mexico. The aerial parts of *Baccharis* species, commonly known as “carqueja”, have many traditional uses in folk medicine, especially for anti-inflammatory, diuretic, and digestive purposes. In addition, infusions or decoctions of *Baccharis articulata* (Lam.) Pers. (*B. articulata*) are also traditionally used as antidiabetic remedies in local folk medicine in southern Brazil [5]. However, as far as we are aware, there are no reports in the literature concerning the hypoglycemic and/or antihyperglycemic properties of this plant.

Therefore, the aim of this study was to investigate the short- and long-term effects of *Baccharis articulata* on glucose homeostasis. The biological effects of the crude extract and the *n*-butanol and aqueous residual fractions were also studied through *in vivo* and *in vitro* approaches. To this end the serum glucose levels, insulin secretion, and muscle and liver glycogen content as well as the intestinal disaccharidase activity and anti-glycation properties were determined. 

## 2. Results and Discussion

### 2.1. Phytochemical Characterization

In our investigation the TLC analysis showed a predominance of phenolic compounds and flavonoids in the crude extract (CE) and the *n*-butanol fraction (BF) of *B. articulata*.This was verified using the Natural Reagent A/UV356 that showed yellow spots corresponding to phenolic compounds (data no shown) as previously described [6]. In addition, it can be observed (Table 1) that the BF showed significantly higher total flavonoids content (44.9 ± 0.71 mg of RE/g of DW, *p* < 0.05) when compared to the CE (38.9 ± 0.66 mg of RE/g of DW) and ARF (25.3 ± 0.42 mg of RE/g of DW). On the other hand the total phenolic content of CE and their related fractions did not shown differences.

Some of the different biological activities of *Baccharis* species are related to the presence of phenolic compounds and flavonoids [7,8]. A large number of studies have demonstrated that phenolic compounds as flavonoids and phenolic acids derivatives have different biological activities, such as antioxidant, anticancer, anti-inflammation and cardioprotective properties, and they can also prevent lipoperoxidation, induce favorable changes in the lipid profile, improve endothelial function, and disclose antithrombotic properties [9]. In addition, the hypoglycemic and/or antihyperglycemic activity of flavonoids has been previously reported [10].

**Table 1:** Total phenolic ^a^ and total flavonoids ^b^ content in crude extract (CE), *n*-butanol fraction (BF) and aqueous residual fraction (ARF) of *B. articulata*.

**Extract/fraction**	**Total phenolic**	**Total flavonoids**
CE	151.8 ± 0.93 ^a^	38.9 ± 0.66 ^a^
BF	154.1 ± 1.08 ^a^	44.9 ± 0.71 ^b^
ARF	135.6 ± 0.95 ^b^	25.3 ± 0.42 ^c^

^a^ Data are mean ± S.E.M. values, expressed as mg of acid gallic equivalents/g of dry weight (n = 6); ^b^ Data are mean ± S.E.M. values, expressed as mg of rutin equivalents/g of dry weight (n = 6); Values in column with the same letters indicate no significant differences (*p* < 0.05).

### 2.2. Effect of Crude Extract and n-Butanol and Aqueous Residual Fractions of *B. Articulata* on Oral Glucose Tolerance Curve

As expected, in the oral glucose tolerance test, after 15 min of glucose loading the glycemia was significantly increased when compared with zero time. Glipizide (100 mg/kg) an oral hypoglycemic agent of the sulfonylurea class was used as a positive control and produced a typical serum glucose lowering at all periods analyzed (15 to 180 min) compared to the hyperglycemic group (Table 2). At all doses tested (50, 100 and 200 mg/kg) the CE of *B. articulata* leaves was effective in reducing the glycemia at different times after oral treatment compared with the respective hyperglycemic control group (Table 2). The dose of 100 mg/kg of the CE produced the best antihyperglycemic profile at 15 to 60 min and the maximum reduction observed was 26% at 30 min. Administration of 50 mg/kg of the BF of *B. articulata* decreased serum glucose levels significantly at 15, 30 and 60 min, and the glycemic reduction was around 23, 23 and 18%, respectively, whereas the dose of 100 mg/kg was not effective in reducing the serum glucose levels during the times studied (Table 2). In addition, oral administration of the ARF of *B. articulata* also reduced the serum glucose levels in hyperglycemic rats at both doses tested (50 and 100 mg/kg) and the antihyperglycemic effect was better with the lower dose. The reduction was around 15 and 20% at 15 and 30 min after treatment, respectively. At 180 min, glycemic levels were similar to the respective results for the hyperglycemic control groups.

On the other hand, the CE, BF and ARF (100 mg/kg) of *B. articulata* were studied in rats with induced diabetes and no significant changes in the serum glucose levels in an acute treatment were observed (data not shown). These results point a potential insulin secretagogue effect for *B. articulata* compounds.

Another *Baccharis* species, *Baccharis trimera* reportedly has potential antidiabetic activity. Oliveira *et al.* [11] investigated the effect of its extracts and fractions on glycemia in non-diabetic mice and mice with streptozotocin-induced diabetes. After 7 days of treatment, the aqueous fraction (2,000 mg/kg, twice daily) reduced the glycemia of diabetic mice. However, in contrast to our results for *B. articulata*, none of the extracts or fractions (200 or 2,000 mg/kg) of *B. trimera* induced any effect on glycemia after acute administration on hyperglycemic mice. Thus, to the best of our knowledge, our results represent the first report of the potential antihyperglycemic effect of *B. articulata.*

Diterpenoids, flavonoids and other phenolic compounds have been reported as the major phyto-constituents of the *Baccharis* species and this diverse chemical composition is related to a variety of biological activities described for these species [8]. The results reported herein demonstrate that *B. articulata* has a significant content of flavonoids and other phenolic compounds (Table 1) and the presence of these constituents may be associated with the antihyperglycemic effect observed, since the hypoglycemic activity of phenolics compounds has been previously reported [10].

### 2.3. Effect of Crude Extract and n-Butanol and Aqueous Residual Fractions of *B. Articulata* on Insulin Secretion and Glycogen Content

In order to evaluate the possible mechanism of action of the extract (CE) and fractions (BF and ARF) of *B. articulata*, their effects on glycogen content and on insulin secretion were investigated. Serum insulin levels were determinated in fasted rats after an oral glucose loading (4 g/kg) as shown in Table 3. As expected a sulfonylurea agent, glipizide, stimulated the insulin secretion by 295, 149 and 191% at 15, 30 and 60 min, respectively, compared to the hyperglycemic control group. The CE potentiated insulin secretion induced by glucose at 15 (167%), 30 (141%) and 60 min (268%), after oral treatment. In addition, the BF increased significantly the insulin secretion by 162, 189 and 244% at 15, 30 and 60 min, respectively. However, the ARF was not able to increase serum insulin levels. The treatments with the CE and BF resulted in around a 2.3-fold increase in the insulinogenic index (II) compared with the hyperglycemic control group (hyperglycemic control 0.44 ng/mg; CE 0.96 ng/mg and BF 1.03 ng/mg), achieving values similar to that calculated for glipizide (1.09 ng/mL). These results indicate, for the first time, the powerful effect of *B. articulata* on insulin secretion.

**Figure 1:**
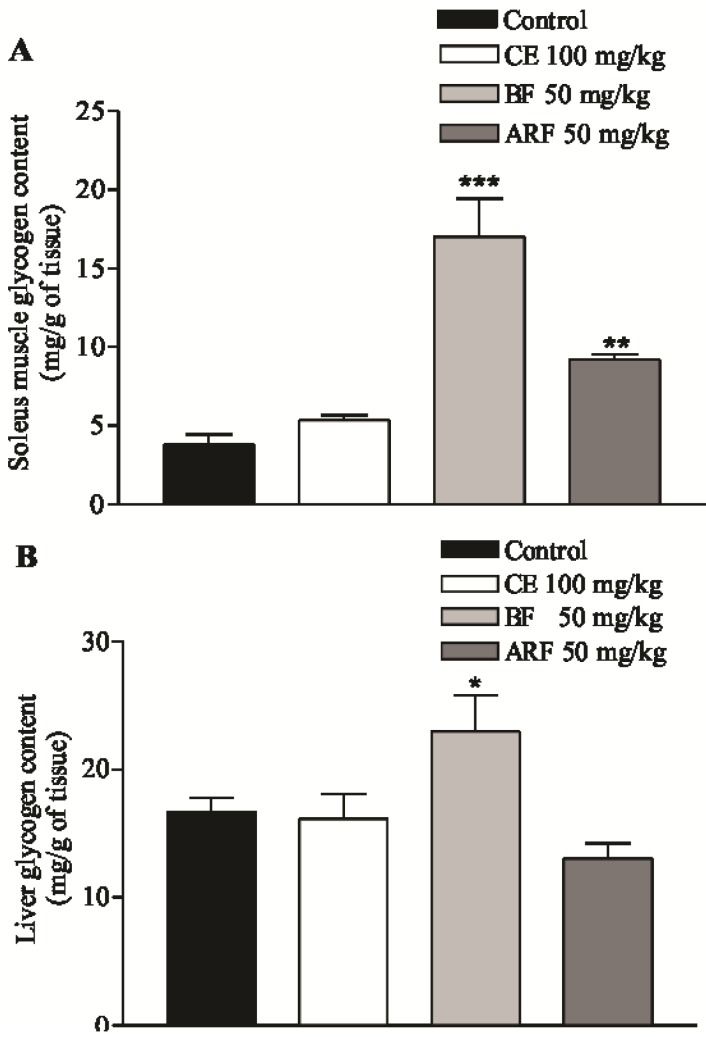
Effect of crude extract (CE), *n*-butanol fraction (BF) and aqueous residual fraction (ARF) of *B. articulata* on glycogen content in comparison to hyperglycemic rats (control). (**A**) soleus muscle and (**B**) liver 3 h after treatment by oral gavage. Values are expressed as mean ± S.E.M; n = 6 in duplicate for each group. Significantly different to the corresponding hyperglycemic group; * *p*< 0.05; ** *p*< 0.001; *** *p*< 0.0001.

The glycogen content in the soleus muscle and liver samples of hyperglycemic rats and those which received acute treatments with the CE (100 mg/kg), BF (50 mg/kg) and ARF (50 mg/kg) was determinate as shown in Figure 1A and B. After 3 h of oral treatment with the BF and ARF the glycogen content in the soleus muscle increased significantly, by around 443 and 212%, respectively, compared to the hyperglycemic control group (Figure 1A). 

**Table 2:** Acute effect of crude extract (CE), *n*-butanol fraction (BF) and aqueous residual fraction (ARF) of *Baccharis articulata* on serum glucose levels (mg/dL) in oral glucose tolerance curve^ a^.

**Time (min)**	**Group I Hyper Glucose (4 g/kg)**	**Group II Hyper + glipizide (10 mg/kg)**	**Group III Hyper + CE**			**Group IV Hyper + BF**		**Group V Hyper + ARF**	
			**50 mg/kg**	**100 mg/kg**	**200 mg/kg**	**50 mg/kg**	**100 mg/kg**	**50 mg/kg**	**100 mg/kg**
0	112 ± 4 ^#^	104 ± 3	110 ± 6	116 ± 5	103 ± 3	116 ± 2	113 ± 3	116 ± 3	103 ± 2
15	162 ± 8	121 ± 4 ***	128 ± 5 **	123 ± 2 ***	134 ± 6 **	124 ± 6 ***	187 ± 4	138 ± 4 **	136 ± 10 *
30	185 ± 6	148 ± 7 ***	144 ± 4 ***	137 ± 1 ***	146 ± 3 ***	143 ± 2 ***	178 ± 6	148 ± 3 ***	159 ± 6 *
60	164 ± 4	122 ± 4 ***	145 ± 5	130 ± 8 **	140 ± 6 *	134 ± 9 **	186 ± 8	154 ± 4	161 ± 10
180	135 ± 4	116 ± 5 **	130 ± 3	129 ± 3	136 ± 4	145 ± 5	126 ± 4	142 ± 5	133 ± 5

^a^ Values are expressed as mean ± S.E.M; n= 6 in duplicate for each treatment; Statistically significant difference compared to the corresponding hyperglycemic group; Statistically significant at ^# ^*p* < 0.001 in relation to 15 min * *p* < 0.05; ** *p* < 0.01; *** *p* < 0.001.

**Table 3:** Acute effect of crude extract (CE), *n*-butanol fraction (BF), aqueous residual fraction (ARF) of *Baccharis articulata* on serum insulin levels (ng/mL) and insulinogenic index (II; ng/mg) ^a^.

**Time (min)**	**Hyper Glucose (4 g/kg)**	**Hyper + glipizide (10 mg/kg)**	**Hyper + *B. articulata***		
			**CE 100 mg/kg**	**BF 50 mg/kg**	**ARF 50 mg/kg**
0	0.57 ± 0.03	-	-	-	-
15	0.77 ± 0.06 ^#^	2.27 ± 0.20 ***	1.29 ± 0.27 **	1.25 ± 0.10 *	0.82 ± 0.04
30	0.90 ± 0.10	1.37 ± 0.02 *	1.30 ± 0.14 *	1.74 ± 0.06 ***	0.79 ± 0.09
60	0.54 ± 0.04	1.03 ± 0.15 *	1.45 ± 0.24 ***	1.32 ± 0.20 ***	0.63 ± 0.10
II	0.44	1.09	0.96	1.03	0.49

^a^ Values are expressed as mean ± S.E.M; n = 4 in duplicate for each treatment; Statistically significant at ^#^ *p* < 0.01 in relation to euglycemic group; Statistically significant difference compared to the corresponding hyperglycemic group; * *p* < 0.05; ** *p* < 0.01; *** *p* < 0.001.

In addition, only the BF treatment was able to significantly increase the glycogen content in the liver when compared with the hyperglycemic control group 3 h after treatment. In percentage terms, this change was 137% (Figure 1B). Taking it in account, it seems that the serum glucose lowering could be related with the effect on increased glycogen content similar to that known to insulin, points to an insulin-mimetic effect.

Insulin is secreted into the bloodstream by β-cells of the endocrine pancreas and glucose is the main insulin secretagogue. Insulin is the most important hormone that regulates energy metabolism and has hypoglycemic effect. An absolute or relative lack of insulin, as in the case of diabetes, leads to severe dysfunction in the major insulin target organs such as muscle, liver and adipose tissue [12].

The stimulation of β-cells and subsequent release of insulin and activation of the insulin receptors is a possible mechanism of natural products with potential antidiabetic activity. Folador *et al.* [13] showed that the crude extract, the *n*-butanol fraction and two isolated *C*-glycosylflavones, isovitexin and swertisin, of *Wilbrandia ebracteata* can have an antihyperglycemic action, which was related to the stimulation of *in vivo* insulin secretion.

Glucose homeostasis is maintained by the balance of liver glucose production and glucose utilization by peripheral tissues. In mammals, glucose is stored as glycogen in the liver and muscle, which are the major sites for glycogen synthesis and storage. It is well known that glycogen deposition from glucose is regulated by insulin. However, it is well reported that flavonoids and plant extracts with proven antihyperglycemic activity can also influence glycogen deposition in different tissues as well as interact with key enzymes of the glycolytic route in rats [14,15].

In addition, it is worth noting that plants and phytochemicals that have a hypoglycemic effect may act by different mechanisms of action to regulate glucose homeostasis, including an increase in insulin secretion from the pancreatic islets (insulin-secretagogue) and/or enhancing or reproducing the effect of insulin (insulin-mimetic) as we demonstrated for *B. articulata*.

### 2.4. Effect of Crude Extract and n-Butanol and Aqueous Residual Fractions of *B. Articulata* on the Disaccharidases

The intestine plays an important role in glucose homeostasis. A therapeutic approach to decreasing postprandial hyperglycemia is to retard the absorption of glucose via inhibition of carbohydrate-hydrolyzing enzymes, such as α-glucosidase, in the intestine. These disaccharidase enzymes are located in the brush border of the small intestine and are required for the breakdown of carbohydrates before monosaccharide absorption. The α-glucosidase inhibitors delay the absorption of ingested carbohydrates, reducing the postprandial glycemia and insulin peaks [16]. Some plants which exhibit properties similar to those of known classes of anti-diabetic drugs, for instance, inhibitors of α-glucosidase such as acarbose, have been identified [17].

**Figure 2:**
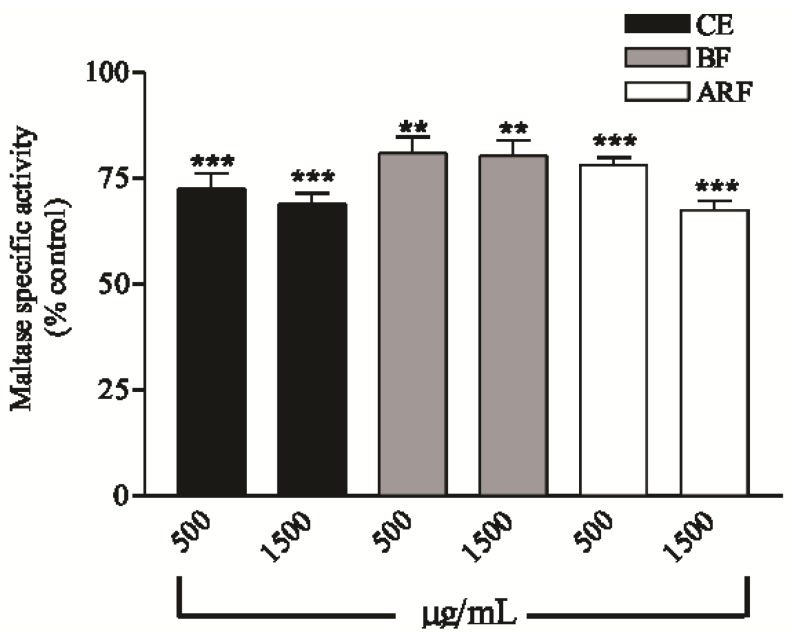
*In vitro* effect of crude extract (CE), *n*-butanol fraction (BF) and aqueous residual fraction (ARF) of *B. articulata* on specific activity of maltase, in the duodenal portion of rat intestine. Incubation = 5 min. Values are expressed as mean ± S.E.M.; n = 6 for each group. Significant at ** *p*< 0.001; *** *p*< 0.0001 compared to control group.

The effect of the extract and fractions of *B. articulata* in disaccharidase assays were determined (Figure 2). A significant effect on maltase inhibition was observed after 5 min of incubation of the intestine homogenate in the presence of a maltose substrate. The CE, BF and ARF were effective at inhibiting the enzyme maltase at both doses tested (500 and 1,500 μg/mL) after 5 min of incubation. 

The inhibitory effect observed ranged from 15 and 32% and the maximum inhibitory effect on maltase activity, above 30%, was observed for the CE and ARF at the higher dose studied, compared with the respective controls. On the other hand, none of the treatments affected the sucrase and lactase activity at any concentration tested (data not shown).

A number of plants are known to exert antihyperglycemic activity through the inhibition of disaccharidase enzymes in the small intestine, impeding the absorption of carbohydrates. Recently, De Souza *et al.* [18] reported that aqueous and methanolic extracts of *B. trimera* efficiently inhibited β and α-glycosidase activity.

### 2.5. Effect of Crude Extract and n-Butanol and Aqueous Residual Fractions of *B. Articulata* on *in vitro* Albumin Glycation

Chronic hyperglycemia and increased oxidative stress during diabetes results in the irreversible formation of advanced glycation endproducts (AGEs), which are a heterogeneous group of molecules formed from non-enzymatic glycation of reducing sugars with free amino groups of proteins, lipids, and nucleic acids. The Schiff’s bases formed by glycation rearrange further through stable reactions to form Amadori products which later, by isomerization, condensation, and rearrangement reactions, form AGEs. The AGEs are known to have a wide range of chemical, cellular, and tissue effects implicated in the development and progression of diabetic complications, like nephropathy, neuropathy, retinopathy, and cardiovascular diseases [19].

**Figure 3:**
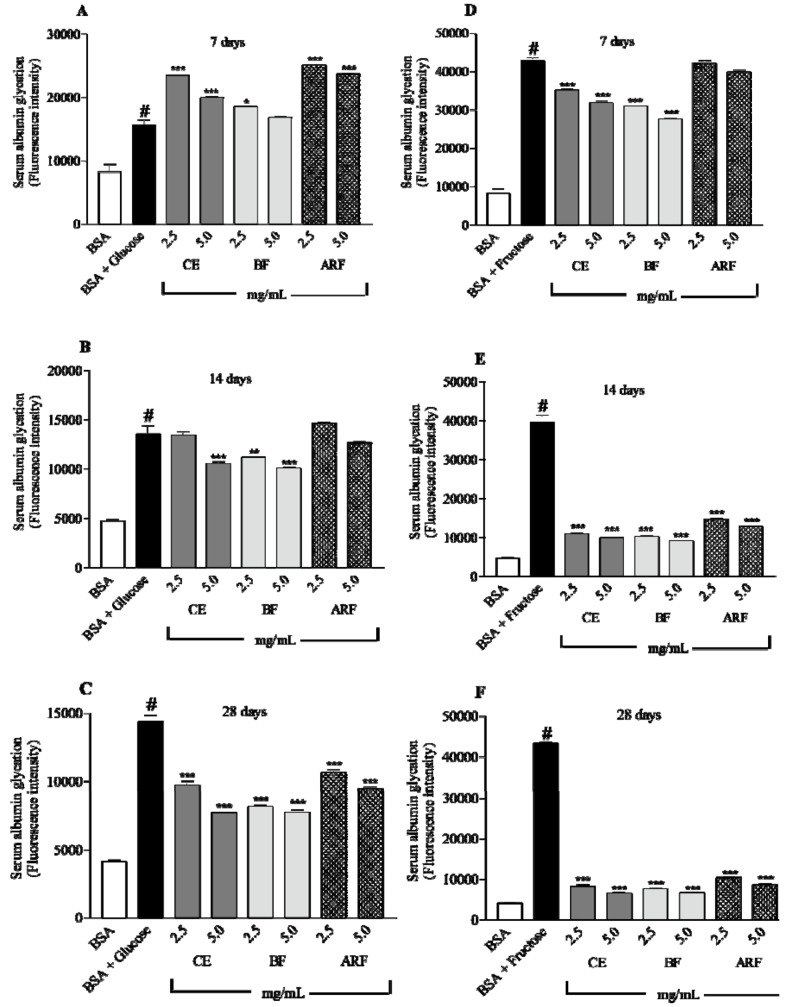
Inhibitory effect of crude extract (CE), *n*-butanol fraction (BF) and aqueous residual fraction (ARF) of *B. articulata* on the formation of fluorescent AGEs in a BSA/glucose or BSA/fructose system. (**A, B and C**) 7, 14 and 28 days BSA/glucose; (**D, E and F**) 7, 14 and 28 days BSA/fructose. Values are expressed as mean ± S.E.M; n = 6 in duplicate for each group. Significantly different to the corresponding control group (BSA/glucose or BSA/fructose); * *p*< 0.05; ** *p*< 0.001; *** *p*< 0.0001.

In the method adopted in this study, BSA was chosen as the model protein and glucose or fructose was used as the glycated agent. This BSA-reducing sugar system is an *in vitro* model widely used in non-enzymatic glycation studies. Proteins can be modified when exposed to reducing sugars through the spontaneous glycation process. The sugar-mediated fluorescence intensity, which is a characteristic of AGEs, increases during incubation at 37 °C for a long period. Figure 3 shows the fluorescence intensity of the products (AGEs) formed in the BSA-glycation model. After incubation at all periods analyzed (7, 14 and 28 days) it was clearly observed that the formation of AGEs was significantly increased in the BSA/glucose (Figure 3A–C) and BSA/fructose (Figure 3D–F) systems when compared with the basal control.

Figure 3B and C show the efficiency of the CE, BF and ARF in the inhibition of albumin glycation with glucose after 14 and 28 days. After 28 days of *in vitro* incubation all treatments caused a glycation reduction of over 30% when compared with positive glycation group (albumin plus glucose). However, a slight increase in glycation was observed for the CE, BF and ARF treatments after 7 days in the BSA/glucose system (Figure 3A). These results show the significant capacity of the extract and fractions of *B. articulata* to reduce the AGE formation after a long period of incubation, the fluorescence intensity being stronger.

The capacity of the CE, BF and ARF to inhibit albumin glycation with fructose for different periods is shown in Figure 3D–F. The reduction in the glycation of albumin by glucose in the presence of the extract and fractions of *B. articulata* increased from 7 to 28 days of treatment. With 7 days of incubation only the CE and BF, at both doses tested, were able to inhibit significantly the albumin glycation. At the maximum period evaluated, more that 75% of glycation reduction was observed when compared with the positive glycation group (albumin plus fructose). 

Several natural compounds have been proposed and tested as inhibitors of glycation and AGE formation, providing additional therapeutic options for the treatment of the various complications associated with diabetes [20]. As the incidence of diabetes continues to rise worldwide, the study of natural products for the treatment and prevention of diabetes, and its associated complications, offers an important opportunity for the development of complementary interventions that may be more acceptable to high-risk populations in the search for non-pharmaceutical alternatives. Our results demonstrate that the extract and fractions of *B. articulata* are potent inhibitors of *in vitro* AGE formation in chronic treatments and this mechanism may help to provide a protective effect against hyperglycemia-mediated protein damage. Recent reports have shown that flavonoids inhibit the formation of AGEs, which is related to their well-known antioxidative effects [21]. In this regard, we verified the high total flavonoid content of *B. articulata*, which may explain the significant AGE inhibition observed for this species.

## 3. Experimental

### 3.1. Materials

Glipizide, glycogen and bovine serum albumin (BSA) were purchased from Sigma-Aldrich Corporation (St. Louis, MO, USA). Glucose, fructose and maltose were purchased from Vetec® AG (Rio de Janeiro, Brazil). All reagents were of analytical grade. Enzyme-linked immunosorbent assay (ELISA) for the quantitative determination of rat insulin (catalogue no. EZRMI-13K) was purchased from Millipore (St Charles, MO, USA). Salts and solvents were purchased from Merck AG (Darmstadt, Germany).

### 3.2. Plant Material

Aerial parts of *Baccharis articulata* (Lam.) Person were collected in Chapecó, State of Santa Catarina, Brazil, in March 2008. The plant material was identified by Prof. Dr. Geraldo Ceni Coelho (Universidade Federal da Fronteira Sul) and a voucher specimen of the plant (ICN 9057) was deposited at the Herbarium of the Botany Department of Universidade Federal do Rio Grande do Sul, Porto Alegre, Brazil.

### 3.3. Preparation of Extract and Fractions of *B. Articulata*

Aerial parts (50 g) of *B. articulata* were crushed and extracted under reflux (90 °C) with 500 mL of ethanol 40 °GL for 30 min. After cooling, each extract was filtered separately, the volume was adjusted to 500 mL with water, and the preparation was separated into two fractions of 250 mL. One fraction was evaporated under reduced pressure to dryness to obtain the crude extract (CE). The ethanol content of the second fraction was removed under reduced pressure, its volume was adjusted to 250 mL with water, and this aqueous suspension was partitioned (3 × 50 mL of *n*-BuOH) yielding the *n*-BuOH (BF) and aqueous residual (ARF) fractions.

### 3.4. Thin-Layer Chromatographic Analysis

Phytochemical profile of *B. articulata* was performed by thin-layer chromatography (TLC) on silica gel plates (Merck F_254_, 20 × 20 cm) using as mobile phase chloroform-ethanol-acetic acid (CHCl_3_:EtOH:HOAc, 60:40:6, v/v) and Diphenylboryloxyethylamine 1% in methanol, (Natural Reagent) as colour reagent [6]. 

### 3.5. Determination of Total Phenolic Content

Total phenolic content of the extract/fractions were determined by the Folin-Ciocalteu assay [22]. Briefly, a 125 µL aliquot of the extract/fractions were assayed with 125 µL of Folin-Ciocalteu reagent. After six min, 1.25 mL of sodium carbonate (20%, wt/vol) was added and the mixture vortex-mixed and diluted with distilled water to a final volume of 2 mL. After 90 min, the absorption was measured at 760 nm, and the total phenolic content was expressed as milligrams of gallic acid equivalents in relation to grams of dry weight (mg of GAE/g DW). All analyses were performed in triplicate.

### 3.6. Determination of Total Flavonoid Content

The total flavonoids content was determined according to Miliauskas *et al.* [23] with minor modifications. Briefly, 1 mL of the extract/fractions at 4 mg/mL was mixed with 1 mL of aluminum trichloride. After 40 min the absorption of each sample (CE, BF and ARF) was measured at 415 nm. The results were expressed as milligrams of rutin equivalents in relation of grams of dry weight (mg of RE/g DW). All analyses were performed in triplicate.

### 3.7. Animals

The male Wistar rats (180–200 g) used in this study were bred in our animal facility and housed in an air-conditioned room (approximately 22 °C) with controlled lighting on a 12:12 h light/dark cycle (lights on from 06:00 to 18:00 h). The animals were maintained with pelleted food (Nuvital, Nuvilab CR1, Curitiba, PR, Brazil), while tap water was available *ad libitum*. Fasted animals were deprived of food for at least 16 h but allowed free access to water. All the animals were monitored and maintained in accordance with the ethical recommendations of the Brazilian Veterinary Medicine Council (CMV) and the Brazilian College of Animal Experimentation (COBEA; Protocol PP00398/CEUA/UFSC).

### 3.8. Oral Glucose Tolerance Curve (OGTC)

Fasted rats were divided into different groups of six animals for each treatment. Group I, hyperglycemic rats that received glucose (4 g/kg; 8.9 M); Group II, rats that received glipizide at a dose of 10 mg/kg; Group III, rats that received the CE at the doses 50, 100 and 200 mg/kg; Group IV, rats the received the BF at doses of 50 and 100 mg/kg; Group V, rats that received the ARF at doses of 50 and 100 mg/kg. The glycemia was measured before the rats received the treatment (zero time). The rats were treated with extract or fraction and loaded with glucose after 30 min and after the glycemia was measured at 15, 30, 60 and 180 min. All treatments were administrated by oral gavage.

### 3.9. Determination of the Plasma Glucose Concentration

Blood samples were collected and centrifuged, and the blood glucose levels were determined by the glucose oxidase method [24].

### 3.10. Insulin Serum Measurements

The insulin levels were measured by enzyme-linked immunosorbent assay (ELISA) according to the manufacturer’s instructions. The range of values detected by this assay was 0.2 ng/mL to 10 ng/mL. The intra- and inter-assay coefficients of variation for insulin were 3.22 and 6.95, respectively, with a sensitivity of 0.2 ng/mL. All insulin levels were estimated by means of colorimetric measurements at 450 nm with an ELISA plate reader (Organon Teknika, Roseland, NJ, USA) by interpolation from a standard curve. Samples were analyzed in duplicate and results were expressed as ng of insulin serum mL^−1^ [14]. The incremental areas under the response curves (AUCs) were calculated. The insulinogenic index (II) was calculated as the ratio between the AUC_insulin_ and AUC_glucose_ (from zero to 60 min) [25].

### 3.11. Glycogen Content Measurements

The soleus muscles and livers were harvested from untreated hyperglycemic rats and from those treated with the CE (100 mg/kg), BF (50 mg/kg) and ARF (50 mg/kg) used for the assay of glycogen content immediately after 3 h of treatment. Glycogen was isolated from these tissues as described by Krisman [26], with minor modifications [27]. The tissues were weighed, homogenized in 33% KOH and boiled at 100 °C for 20 min, with occasional stirring. After cooling, 96% ethanol was added to the samples which were then heated to boiling followed by cooling in an ice bath to aid the precipitation of glycogen. The homogenates were centrifuged at 1,300 × g for 15 min, the supernatant was discarded and the pellets were neutralized with saturated NH_4_Cl before being maintained at 100 °C for 5 min, washed and resolubilized in water. Glycogen content was determined by treatment with iodine reagent and the absorbance was measured at 460 nm. The results are expressed as mg of glycogen/g of tissue.

### 3.12. Disaccharidase Extraction and Assays

A segment of the small intestine was removed, washed in 0.9% NaCl solution, dried on filter paper, weighed, trimmed and homogenized (300 rpm) with 0.9% NaCl (400 mg of duodenum per mL) for 1 min at 4 °C. The resulting extract was centrifuged at 8,000 rpm for 8 min. The supernatant was used for the measurement of *in vitro* maltase, sucrase and lactase activities and for protein determination.

Maltase (EC 3.2.1.20), lactase (EC 3.2.1.23) and sucrase (EC 3.2.1.48) activities were determined using a glucose diagnosis kit based on the glucose oxidase reagent. For the determination of disaccharidase activity, 50 μL of homogenate were pre-incubated at 37 °C for 5 min, in the absence (control) or in the presence of the CE, BF or ARF of *B. articulata* (treated). The concentrations 500 and 1,500 μg/mL were used. The duodenum homogenates were then incubated at 37 °C for 5 min with 25 μL of the substrate (corresponding to 0.056 µM of maltose, sucrose or lactose) [28].

One enzyme unit (U) was defined as the amount of enzyme that catalyzed the release of 1 μmol of glucose per min under the assay conditions. The specific activity was defined as enzyme activity (U) per mg of protein. Protein concentration was determined by the method described by Lowry method [29] using bovine serum albumin as the standard. The assays were performed in duplicate and conducted along with appropriate controls.

### 3.13. Formation of Advanced Glycation End-Products (AGEs) in Bovine Serum Albumin/Glucose and Fructose Systems

AGE was formed in *in vitro* systems using a previously described method [30]. In brief, BSA (10 mg/mL) in phosphate buffered-saline (PBS, pH 7.4) containing 0.02% sodium azide was incubated with glucose (500 mM) or fructose (100 mM) at 37 °C for 14 and 28 days in the absence (control) and presence of the CE, BF or ARF of *B. articulata* (2.5 and 5.0 µg/mL). The protein, glucose or fructose, and the prospective inhibitor were simultaneously introduced into the incubation mixture. Each solution was kept in the dark in a capped vial, and incubation was allowed to proceed in triplicate vials. In the time-course experiments on AGE formation, we measured characteristic fluorescence (excitation wavelength of 370 nm and emission wavelength of 440 nm) with an Infiniti M200 (TECAN).

### 3.14. Data and Statistical Analysis

Data were expressed as means ± S.E.M. One-way analysis of variance (ANOVA) followed by the Bonferroni *post-hoc* test or unpaired Student’s *t*-test to determine significant differences between the groups. Differences were considered to be significant at *p* < 0.05. 

## 4. Conclusions

In conclusion, the results reported herein indicate that *B. articulata* possesses antihyperglycemic potential. The *n*-butanol fraction, with the highest flavonoids content, showed an important insulin secretagogue effect, exhibiting an insulinogenic index similar to that of glipizide, and also contributed significantly to the storage of glucose as glycogen in muscle and liver tissue. Additionally, the extract and fractions of *B. articulata* demonstrated an inhibitory *in vitro* effect on maltase activity and AGE formation, resulting in short and long-term influence on the decrease in glucose absorption and the prevention of protein glycation. Overall, the results of this study suggests that *B. articulata* has a beneficial *in vivo* and *in vitro* biological effect on glucose homeostasis, which may ameliorate significantly diabetes mellitus status.
